# The longitudinal relationship between fear of movement and physical activity after cardiac hospitalization: A cross lagged panel model

**DOI:** 10.1371/journal.pone.0297672

**Published:** 2024-04-03

**Authors:** Paul Keessen, Kees Jan Kan, Gerben ter Riet, Bart Visser, Harald T. Jørstad, Corine H. M. Latour, Ingrid C. D. van Duijvenbode, Wilma J. M. Scholte op Reimer

**Affiliations:** 1 Faculty of Health, Centre of Expertise Urban Vitality, Amsterdam University of Applied Sciences, Amsterdam, the Netherlands; 2 Research Institute of Child Development and Education, University of Amsterdam, Amsterdam, the Netherlands; 3 Department of Cardiology, Amsterdam University Medical Centre, Amsterdam, the Netherlands; 4 Research Group Chronic Diseases, Utrecht University of Applied Sciences, Utrecht, the Netherlands; Ningbo University, CHINA

## Abstract

**Background:**

Little is known about the association between fear of movement (kinesiophobia) and objectively measured physical activity (PA), the first 12 weeks after cardiac hospitalization.

**Purpose:**

To assess the longitudinal association between kinesiophobia and objectively measured PA and to assess the factor structure of kinesiophobia.

**Methods:**

We performed a longitudinal observational study. PA was continuously measured from hospital discharge to 12 weeks using the Personal Activity Monitor. The PAM measures time spent per day in PA-intensity categories: light, moderate and heavy. Kinesiophobia was assessed with the Tampa Scale for Kinesiophobia (TSK) at four time points (hospital discharge, 3, 6 and 12 weeks). The longitudinal association between PA-intensity and kinesiophobia was studied with a random intercept cross lagged panel model (RI-CLPM). A RI-CLPM estimates effects from kinesiophobia on objectively measured PA and vice versa (cross-over effects), and autoregressive effects (e.g. kinesiophobia from one occasion to the next).

**Results:**

In total, 116 patients (83.6% male) with a median age of 65.5 were included in this study. On no occasion did we find an effect of kinesiophobia on PA and vice versa. Model fit for the original model was poor (X^2^: = 44.646 P<0.001). Best model fit was found for a model were kinesiophobia was modelled as a stable between factor (latent variable) and PA as autoregressive component (dynamic process) (X^2^ = 27.541 P<0.12).

**Conclusion:**

Kinesiophobia and objectively measured PA are not associated in the first 12 weeks after hospital discharge. This study shows that kinesiophobia remained relatively stable, 12 weeks after hospital discharge, despite fluctuations in light to moderate PA-intensity.

## Introduction

After (acute) cardiac hospitalization, only 17% of patients perform the recommended amount of weekly physical activity (PA) which constitutes: 150–300 minutes of moderate-intensity aerobic PA or 75–150 minutes of vigorous intensity aerobic PA [1, 2]. Physical activity is defined as ‘any bodily movement produced by skeletal muscles that results in energy expenditure’ [3]. One potential explanation for these low PA-levels might be kinesiophobia (fear of movement).

Kinesiophobia is described as an irrational, debilitating fear of movement and is explained by the fear avoidance model (FAM) [4]. The FAM is a biobehavioral model which describes how individuals develop avoidance behavior based on pain related fear.

In patients with coronary artery disease (CAD), the prevalence of kinesiophobia varies from 45–75% after hospitalization to 20% after three months. In addition, a longitudinal study revealed that high levels of kinesiophobia are present in 21.1% after 4 months [5]

High levels of kinesiophobia are associated with long disease duration, decreased muscle strength and reduced quality of life [6–8]. In addition, kinesiophobia is associated with low self-efficacy and maladaptive coping strategies, which in turn impede movement behavior [9–11].

Although kinesiophobia is associated with disability, low self-efficacy, and self-reported PA [12–14], the relation between kinesiophobia and objective measures of PA is less clear. The longitudinal association between kinesiophobia and daily PA, measured with an accelerometer, has not been prospectively investigated. Using an accelerometer allows for objective measurement of patients daily PA, instead of subjective methods which are prone to recall bias [15]. Better understanding of the longitudinal association between kinesiophobia and objectively measured PA is necessary to gain insight in the concept of kinesiophobia and may inform the development of future treatment strategies.

The aim of this study was to explore the longitudinal relationship between kinesiophobia and objectively measured physical activity in the first phase (12 weeks) after hospital discharge, in patients with cardiovascular disease.

## Materials and methods

**Fig 1:**
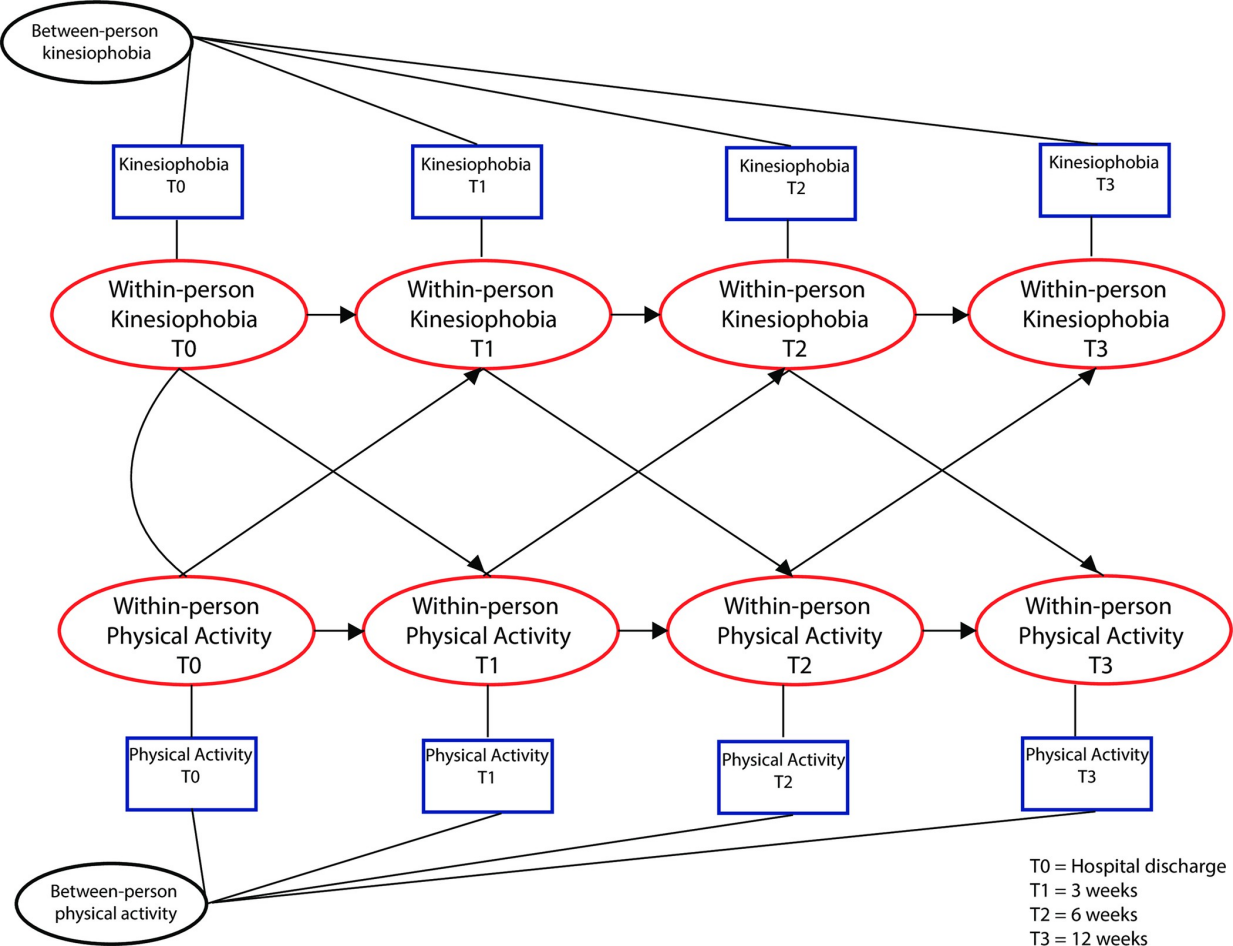
Hypothetical random intercept cross lagged panel model.

In this prospective observational study, we used a cross-lagged panel model (CLPM) to explore the longitudinal association between kinesiophobia and objectively measured PA. CLPM’s are a type of discrete time, structural equation model, used to analyze panel data in which two or more variables are both measured at two or more points in time. CLPM’s aim to estimate the effects of one variable on another at different points in time [16, 17]. In order to account for stable, trait-like differences between patients, such that the lagged relations pertain exclusively to within patient fluctuations, a random intercept is added to the model (RI-CLPM) at each point time. To fit a RI-CLPM, data is decomposed into within-patient dynamics and stable between patient differences, where the latter, account for unobserved heterogeneity [18]. **(Fig 1)**.

### Sample size consideration

For this study four times points (T = 4) were used. Given the exploratory nature of this study, a sample size of N = 100 was deemed sufficient to reach model convergence [19].

### Patient population

Eligible patients had been hospitalized for acute coronary syndrome (ACS), stable angina pectoris (AP), acute heart failure (AHF) or atrial fibrillation (AF) and had undergone a percutaneous coronary intervention (PCI) or electro-cardioversion (ECV). All patients gave written informed consent before participating. Patients were excluded if they were unable to perform physical activity, wear an accelerometer or fill in questionnaires (e.g. due to language problems), or transferred to a nursing home.

### Data collection and measurements

Patients were identified, between August 2019 and May 2021, through the electronic health records system of the Amsterdam University Medical Centre. In the hospital, eligible patients were approached by researchers of the Amsterdam University of Applied Sciences and enrolled in this study. The following data were collected from the electronic health records: age, sex, education, marital status, cardiac diagnosis and disease history. Patients were asked by email to complete a questionnaire about kinesiophobia at four time points: at hospital discharge (week 1), 3, 6 and 12 weeks post-discharge. In addition, patients were asked to wear an accelerometer (see below) to assess PA-levels for 12 weeks directly after hospital discharge.

### Outcomes

#### Kinesiophobia

Patients completed the Tampa Scale for Kinesiophobia (TSK-NL Heart). The TSK-NL Heart consists of 13 questions, each on a four-point scale ranging from 1 to 4, with total scores ranging between 13 and 52. Scores on the TSK-NL Heart are categorized as follows: subclinical: 13–22; mild: 23–32; moderate: 33–42; and severe: 43–52. The TSK-NL Heart has substantial internal consistency (Chronbach’s α: 0.88), test-retest reliability (ICC: 0.80, 95% CI 0.72 to 0.85) and correlates reasonably well to the Cardiac Anxiety Questionnaire (CAQ) (R ^*spearman*^: 0.61, (95% CI 0.51 to 0.71) and the Hospital Anxiety and Depression Scale (Anxiety) (R ^*spearman*^: 0.60 95% CI: 0.48 to 0.70) [7]. In this study the TSK-NL Heart was analyzed as continuous variable.

#### Objectively measured physical activity

At hospital discharge, patients were asked to wear a PAM-AM300 © Personal Activity Monitor (PAM) at waist level (belt, trouser or pocket) during all daily activities. The PAM is an uniaxial accelerometer and measures PA intensity by calculating the time spent doing light, medium or heavy PA. Categories of PA are based on metabolic equivalents of tasks (MET’s) and are multiplies of the resting metabolism, reflecting the metabolic rate during PA. Light PA comprise activities such as cooking and doing groceries (<3 MET), moderate PA comprise activities such walking or cycling (3–6 MET) and heavy PA comprise activities as aerobics, running or cycle racing (>6 MET) [20].

The PAM is a validated tool to assess physical activity intensity in patients with various conditions [21]. The PAM has substantial reliability (ICC: 0.80; 95% CI: 0.28 to 0.92) and significantly correlates with the Actigraph accelerometer (R ^*spearman*^: 0.82). Patients were asked to wear the PAM-sensor for 90 days. Scores are presented as minutes of PA per day/per week, in the categories light, moderate, heavy and total activity.

### Data analysis

#### Descriptive statistics

Descriptive data were presented as frequencies (proportion), mean (SD) or median (IQR). Associations between physical activity and kinesiophobia were analyzed with correlational analysis using the spearman rank correlation coefficient. Magnitude of the association was interpreted as small (0.00 to 0.30), medium (>0.30 to 0.50) and large (>0.50) [22]. Differences in kinesiophobia and objectively measured PA between hospital discharge and 12 weeks follow up were assessed with the Wilcoxon signed rank test.

#### The basic RI-CLPM

For this study, TSK-NL Heart scores and total physical activity scores (average minutes per day/per week) of week 1 (hospital discharge), 3, 6 and 12 after hospital discharge were used. Observed scores (kinesiophobia and PA) were decomposed into grand means, stable *between*-components and fluctuating *within*-components. This model is illustrated in **Fig 1**. In this model, the blue squares represent the observed kinesiophobia and PA scores. The *between*-components (random intercepts) of kinesiophobia and PA capture the persons’ deviations from the grand mean and represent stable differences between patients. The random intercepts are specified as a latent variable with the repeated measures as its indicators, fixing all factor loadings to 1. The *within-*components (red ovals) are the differences between a patient’s observed and expected score, based on the grand means and its random intercepts. The following structural relations between within components were specified: firstly: *Autoregressive effects* (e.g. from T0-kinesiophobia at T1-kinesiophobia) represent the within carry-over effect from one occasion to the next. If this effect is positive, this implies that an individual who experiences elevated kinesiophobia at time = t, relative to his/her own score, is likely to experience kinesiophobia at time = t+1. Secondly, *The cross-lagged effects* represent the cross-over effects from one domain to the other (e.g. T0-kinesiophobia to T1-PA). A positive effect implies that deviation from an individual’s level of kinesiophobia will likely be followed by a positive deviation in PA. Autoregressive and cross-lagged effects are presented as standardized beta effect size estimates (β). Effect sizes were interpreted as small (<0.29), moderate (0.30–0.49), large (≥ 0.50) [22]. Model fit was assessed with Chi square test for model fit (X^2^), Comparative Fit Index (CFI) and Tucker Lewis Index (TLI). Model fit was deemed acceptable if X^2^ p >0.05, and CFI/TLI >0.95 [23]. Missing values of the TSK-NL Heart were: Hospital discharge: 34 (22.8%), 3 weeks: 37 (24.8%), 6 weeks: 42 (28.2%), 12 weeks: 54 (36.2%). Of the 149 patients, 33 (22.1%) did not wear the PAM accelerometer. In total, 116 patients were included in the final sample. In the PAM accelerometer data, the total amount of missing data was 14.4%. Little’s MCAR test was used to determine patterns of missing data. (Little’s MCAR Test Chi Square = 4871,310 DF = 4995, Sig = 0.893). Missing data were handled using Full Conditional Specification Multiple imputation (FCS-MI). Multiple imputation and descriptive statistics were performed in SPSS V28 and the RI-CLPM was performed in Mplus V8.

## Results

### Demographic and clinical characteristics

**Fig 2:**
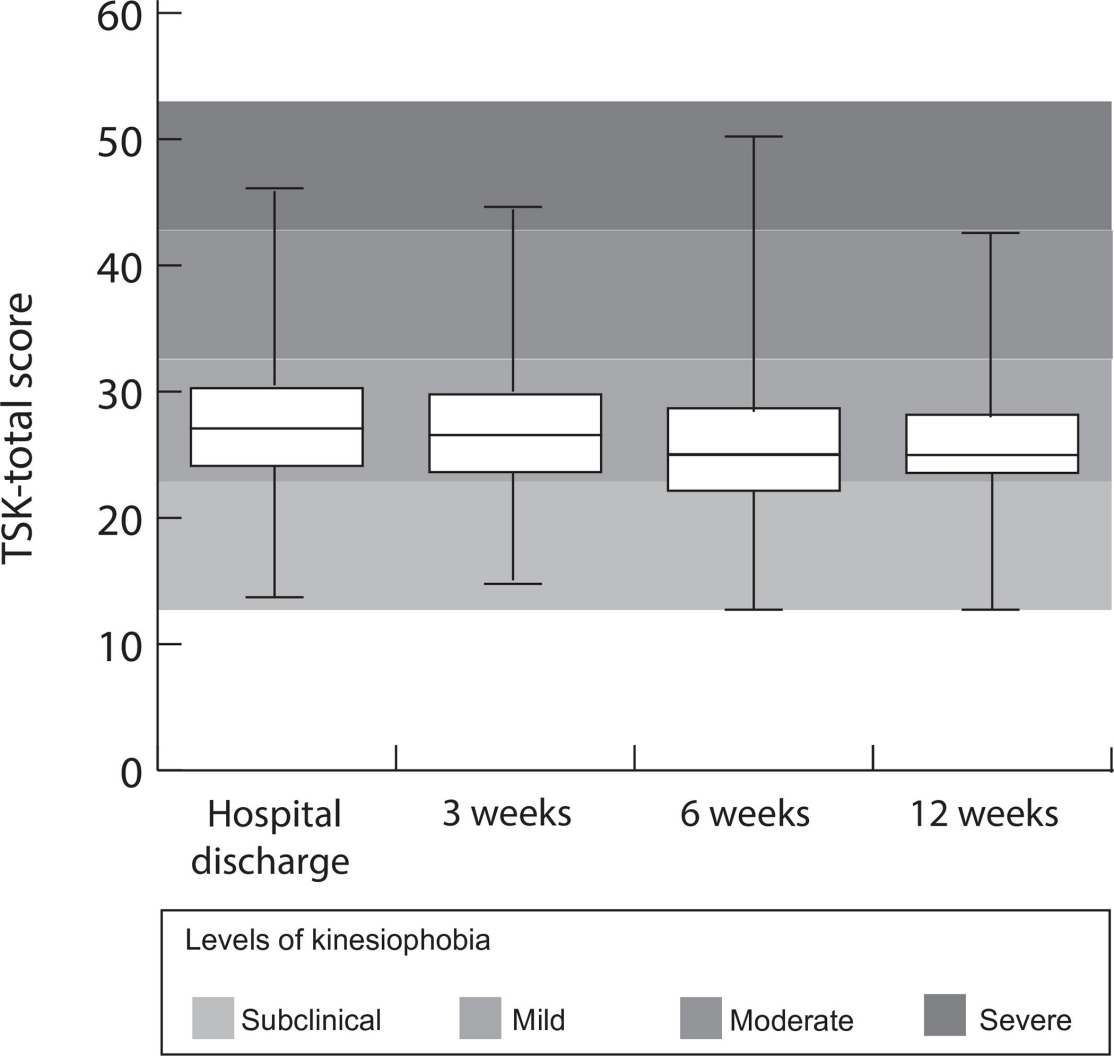
Kinesiophobia at hospital discharge, 3,6 and 12 weeks after hospital discharge (N = 116).

In total, 188 patients were assessed for eligibility. After inclusion, two patients died and 149 patients completed the TSK-Heart NL questionnaires. Of these patients, 33 did not wear the PAM accelerometer. Finally, 116 patients were included in the analyses with a median (IQR) age of 65.5 (15) years. The majority of patients were male (83.6%) and lived with a partner (78.4%). Most patients had been admitted for an elective intervention (53.4%) and had undergone PCI (81%). In this study, 45 (38,8%) initiated cardiac rehabilitation (CR). Those that initiated CR had lower levels of light PA (at T0 and T1) compared to those that did not initiate CR. **(Table 1)**. An overview of kinesiophobia levels at each time point is presented in **Fig 2**.

**Table 1:** Population characteristics.

(N = 116)							
**Demographics**							
Age, years, median (IQR)			65.5 (9.88)				
Male (%)			97 (83.6)				
Ethnicity (%)							
*White*			107 (92.2)				
*Black*			2 (1.7)				
*Asian*			7 (6.0)				
Education (%)							
*No education*			6 (5.2)				
*Lower education*			25 (21.6)				
*Middle education*			46 (39.6)				
*Higher education*			39 (33.6)				
Lives with partner (%)			91 (78.4)				
BMI category (*kg/m*^*2*^)							
*18–25*			15 (12.9)				
*25–30*			92 (79.3)				
*>30*			9 (7.8)				
**Index event (%)**							
Acute Coronary Syndrome							
*STEMI*			25 (21.6)				
*NSTEMI*			19 (16.4)				
*UAP*			9 (7.8)				
Stable Angina revascularization			44 (37.9)				
Acute Heart Failure			1 (0.9)				
Atrial Fibrillation			18 (15.5)				
**Admission type (%)**							
Acute			54 (46.6)				
Elective			62 (53.4)				
**Treatment for index event (%)**							
PCI			94 (81.0)				
ECV			16 (13.8)				
Medication only			6 (5.2)				
**Cardiac disease history (%)**							
Myocardial infarction			27 (23.3)				
PCI			43 (37.1)				
CABG			4 (3.4)				
Stroke			11 (9.5)				
**Kinesiophobia (TSK-NL Heart, 13–52) (%)**							
	*Subclinical*		*Mild*		*Moderate*		*Severe*
Hospital Discharge (T0)	28 (24.1)		64 (55.2)		23 (19.1)		1 (0.9)
3 weeks (T1)	25 (21.6)		69 (59.5)		20 (17.2)		2 (1,7)
6 weeks (T2)	38 (32.8)		61 (52.5)		14 (12.2)		3 (2.6)
12 weeks (T3)	31 (26.7)		73 (62.9)		11 (9.5)		1 (0.9)
**Physical activity (PA), Median (IQR)**							
	*Light PA*		*Moderate PA*		*Heavy PA*		*Total PA*
*Timepoint*							
Hospital Discharge (T0)	54.14(32.04)		24.34 (24.10)		0.43 (1.32)		82.09 (53.98)
3 weeks (T1)	82.86 (48.72)		39.21 (27.43)		0.57 (1.40)		127.50 (60.96)
6 weeks (T2)	84.04 (35.89)		35.50 (28.82)		0.83 (1.71)		127.00 (59.21)
12 weeks (T3)	81.34 (28.36)		40.15 (16.11)		1.15 (2.22)		123.85 (39.57)
**Variable**		**Hospital discharge (T0)**		**3 months (T3)**		**P-value**	
TSK total score median (IQR)		27.43 (7.28)		25.00 (5.90)		0.002	
Light PA median (IQR)		54.14 (32.04)		81.34 (28.86)		0.001	
Medium PA median (IQR)		23.34 (24.10)		40.15 (16.11)		0.001	
Heavy PA median (IQR)		0.43 (1.32)		1.15 (2.22)		0.001	
**Variable**		**CR initiation No (N = 71)**		**CR initiation Yes (N = 45)**		**P-value**	
TSK T0 median (IQR)		27.45 (10.00)		27.37 (6.68)		0.80	
TSK T3 median (IQR)		25.20 (5.13)		24.54 (6.00)		0.25	
Light PA T0 median (IQR)		58.50 (36.86)		47.33 (30.01)		0.02*	
Light PA T3 median (IQR)		81.35 (26.41)		77.71 (24.46)		0.04*	
Medium PA T0 median (IQR)		26.15 (26.66)		19.14 (22.06)		0.06	
Medium PA T3 median (IQR)		40.15 (19.57)		39.00 (12.25)		0.32	
Heavy PA T0 median (IQR)		0.57 (1.42)		0.43 (0.86)		0.72	
Heavy PA T3 median (IQR)		1.18 (2.22)		0.86 (2.07)		0.96	
**Variable**		**Acute (N = 54)**		**Elective (N = 62)**			
TSK T0 median (IQR)		27.71 (6.09)		27.33 (11.00)		0.98	
TSK T3 median (IQR)		24.84 (5.09)		25.41 (7.00)		0.21	
Light PA T0 median (IQR)		51.79 (26.96)		56.41 (33.09)		0.20	
Light PA T3 median (IQR)		81.35 (38.14)		81.35 (22.29)		0.78	
Medium PA T0 median (IQR)		19.57 (22.79)		26.15 (27.08)		0.12	
Medium PA T3 median (IQR)		39.21 (19.27)		40.15 (18.20)		0.12	
Heavy PA T0 median (IQR)		0.42 (1.00)		0.61 (1.28)		0.11	
Heavy PA T3 median (IQR)		0.86 (2.22)		1.94 (2.22)		0.27	

Abbreviations: STEMI = ST-Elevation Myocardial Infarction, NSTEMI = Non ST-Elevation Myocardial Infarction, UAP = Unstable Angina Pectoris, PCI = Percutaneous Coronary Intervention, ECV = Electro Cardio Version, CABG = Coronary Artery Bypass Grafting. Acute admission = Unplanned hospital admission, Elective admission = Planned admission, Lower education = VMBO preparatory middle-level applied education. Middle education = Mid-level education, Higher general continued education, Higher education = preparatory scientific education, University of Applied Sciences or higher CR = Cardiac Rehabilitation. CR-initiation = Started with Cardiac Rehabilitation.

**Fig 3:**
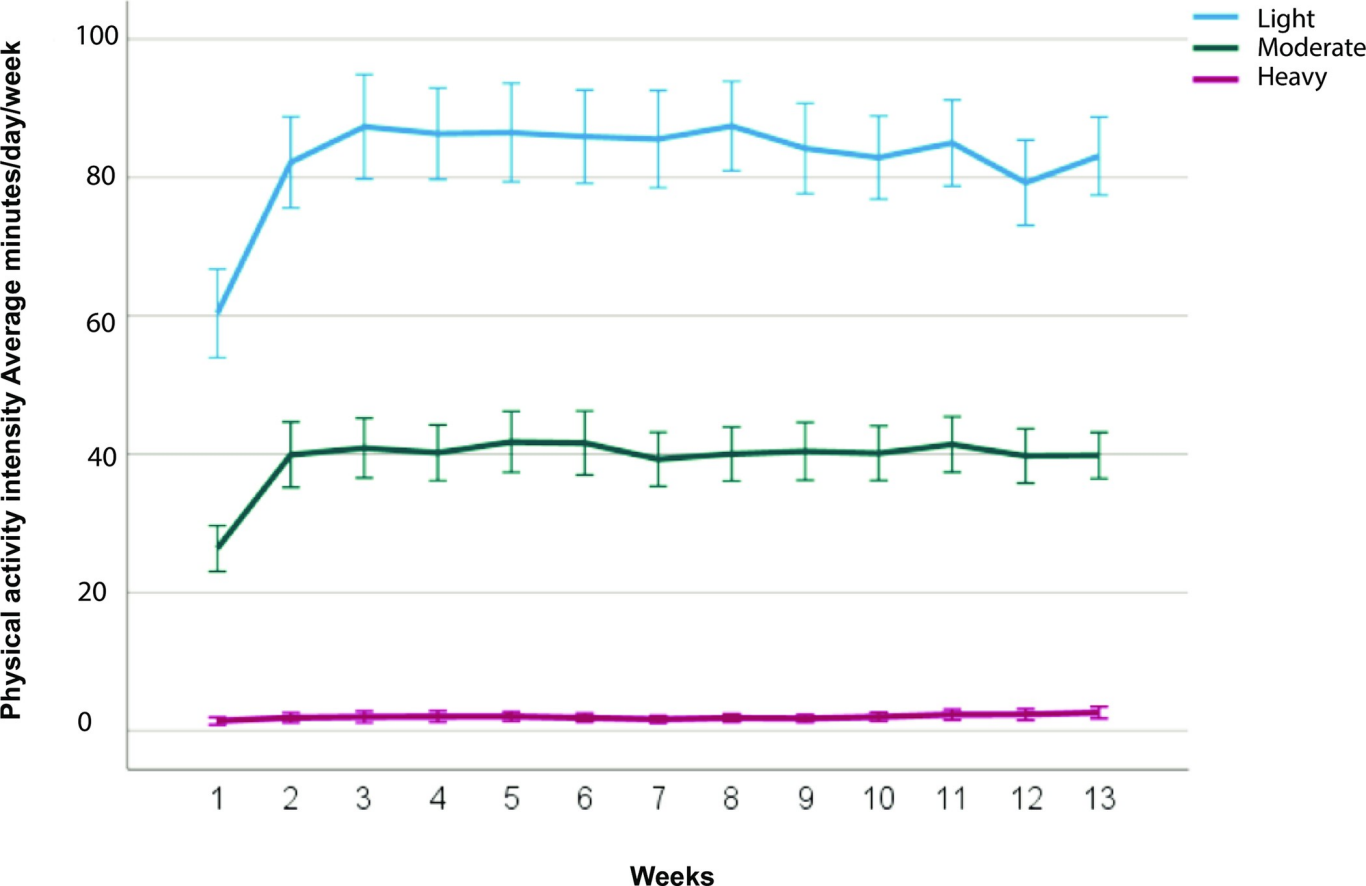
Physical activity scores (per day/per week) measured with the Personal Activity Monitor (PAM) (N = 116).

Median TSK scores (IQR) decreased from week 1: 27.4 (7.28), to 12 weeks: 25 (5.90) (P<0.002). Median light activity (minutes/day/per week) (IQR) improved from week 1: 54.14 (32.04) to 12 weeks: 81.34 (28.36) (P<0.001). Median medium activity (minutes/day/per week) (IQR) improved from week 1: 24.34 (24.10) to 12 weeks: 40.15 (16.11) (P<0.001). Median heavy activity (minutes/day/per week) (IQR) improved from week 1: 0.43 (1.32) to 12 weeks: 1.49 (2.21) (P<0.001) **(Fig 3)**.

### Correlation matrix

**Table 2** shows all correlations between Kinesiophobia (TSK- scores) and Objectively measured PA. Small positive correlations were found between TSK-week 1 and Moderate PA-week12: R ^*spearman*^: 0.21 (95% CI: 0.01 to 0.39), TSK-week 6 and Moderate PA-week 3: R ^*spearman*^: 0.22 (95%CI: 0.03 to 0.40), TSK-week 6 and Heavy PA WK 3: R ^*spearman*^: 0.19 (95% CI: 0.02 to 0.40), and TSK-week 6 and Heavy PA-week 6: R ^*spearman*^: 0.23 (95%CI: 0.04 to 0.40).

**Table 2:** Correlational analyses between kinesiophobia (TSK) and Physical activity (PA).

	TSK week 1	TSK week 3	TSK week 6	TSK week 12
Light PA week 1	-.046	.090	.096	-.010
Light PA week 3	.103	-.005	.114	-.051
Light PA week 6	-.003	.066	.077	-.057
Light PA week 12	.022	.036	.049	.037
Moderate PA week 1	.000	.047	.127	-.014
Moderate PA week 3	.135	.034	**.221** ^*^	.097
Moderate PA week 6	.114	.112	.173	.096
Moderate PA week 12	.210^*^	.034	.061	.078
Heavy PA week 1	-.083	-.028	.098	.025
Heavy PA week 3	.016	-.010	**.191** ^*^	.102
Heavy PA week 6	.061	.121	**.233** ^*^	.128
Heavy PA week 12	.189^*^	.080	.108	**.229** ^*^

* Correlation is significant at the level of <0.05 (2-tailed)

PA = Physical activity

TSK = Tampa Scale for Kinesiophobia

### Random intercept cross lagged panel model

**Fig 4:**
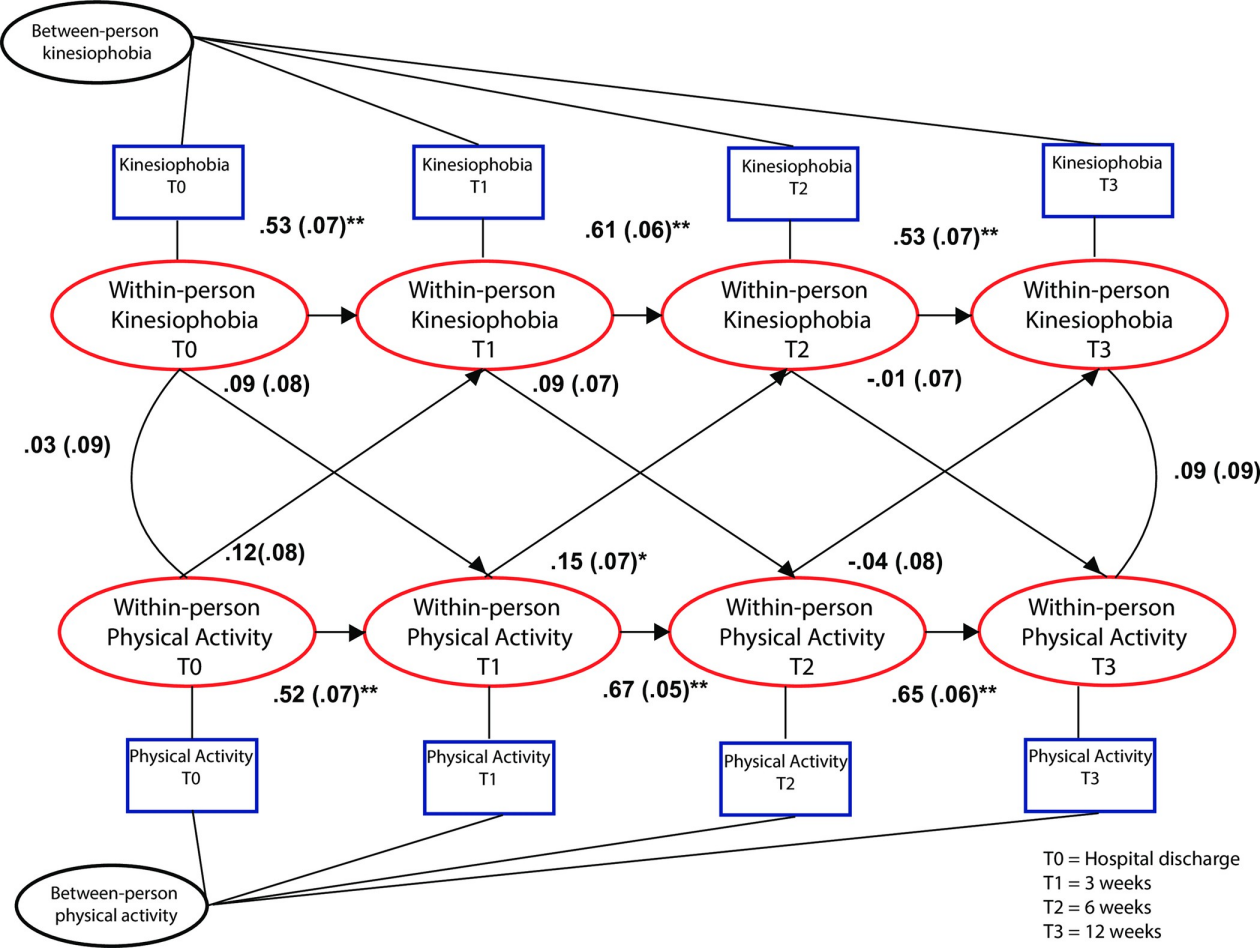
Random intercept cross lagged model with kinesiophobia and objectively measured physical activity.

Large statistically significant autoregressive effects (β > 0.5) for kinesiophobia and total PA were found at all occasions, indicating the presence of large carry-over effects from one occasion to the next. Patients who had elevated levels of total PA at time = t were also likely to have elevated levels of total PA at time = t+1. Similar carry-over effects were found for total PA. One small cross-over effect was found from Total PA-week1 to TSK-Week3: β = 0.15 (95%CI: 0.01 to 0.29) indicating that total physical activity at three weeks was associated with increased kinesiophobia at 6 weeks. Model fit for this model was poor (Chi Square = 44.646 P<0.001) **(Table 3)**. This model is presented in **Fig 4**.

**Table 3:** Random intercept cross lagged panel model.

	Standardized Beta (95% CI)	S.E.	EST./S.E.	Two Tailed P-value
*Cross lagged and autoregressive effects*				
**Independent variable**	**Dependent variable**			
	**TSK week 3**			
TSK week 1	0.53 (0.40 to 0.66)	0.067	7.981	<0.000
PA week 1	0.12 (-0.03 to 0.27)	0.078	1.521	<0.128
	**TSK week 6**			
TSK week 3	0.61 (0.50 to 0.73)	0.057	10.755	<0.000
PA week 3	0.15 (0.01 to 0.29)	0.071	2.120	<0.034
	**TSK week 12**			
TSK week 6	0.53 (0.39 to 0.66)	0.068	7.767	<0.000
PA week 6	-0.04 (-0.19 to 0.12)	0.080	-0.449	<0.653
	**PA week 3**			
TSK week 1	0.09 (-0.6 to 0.25)	0.079	1.190	<0.234
PA week 1	0.52 (0.39 to 0.65)	0.068	7.663	<0.000
	**PA week 6**			
TSK week 3	0.09 (-0.04 to 0.23)	0.068	1.356	<0.175
PA week 3	0.67 (0.58 to 0.77)	0.050	13.348	<0.000
	**PA week 12**			
TSK week 6	-0.01 (-0.15 to 0.13)	0.071	-0.182	<0.856
PA week 6	0.65 (0.54 to 0.76)	0.055	11.851	<0.000
*Model fit*				
Chi Square = 44.646 P<0.001				
CFI = 0.88				
TLI = 0.74				
DF = 12				

TSK = Tampa Scale for Kinesiophobia

PA = Total physical activity (light + moderate + heavy) (per day/per week)

DF = Degrees of freedom

### Alternative model: Random intercept model without cross lagged effects

**Fig 5:**
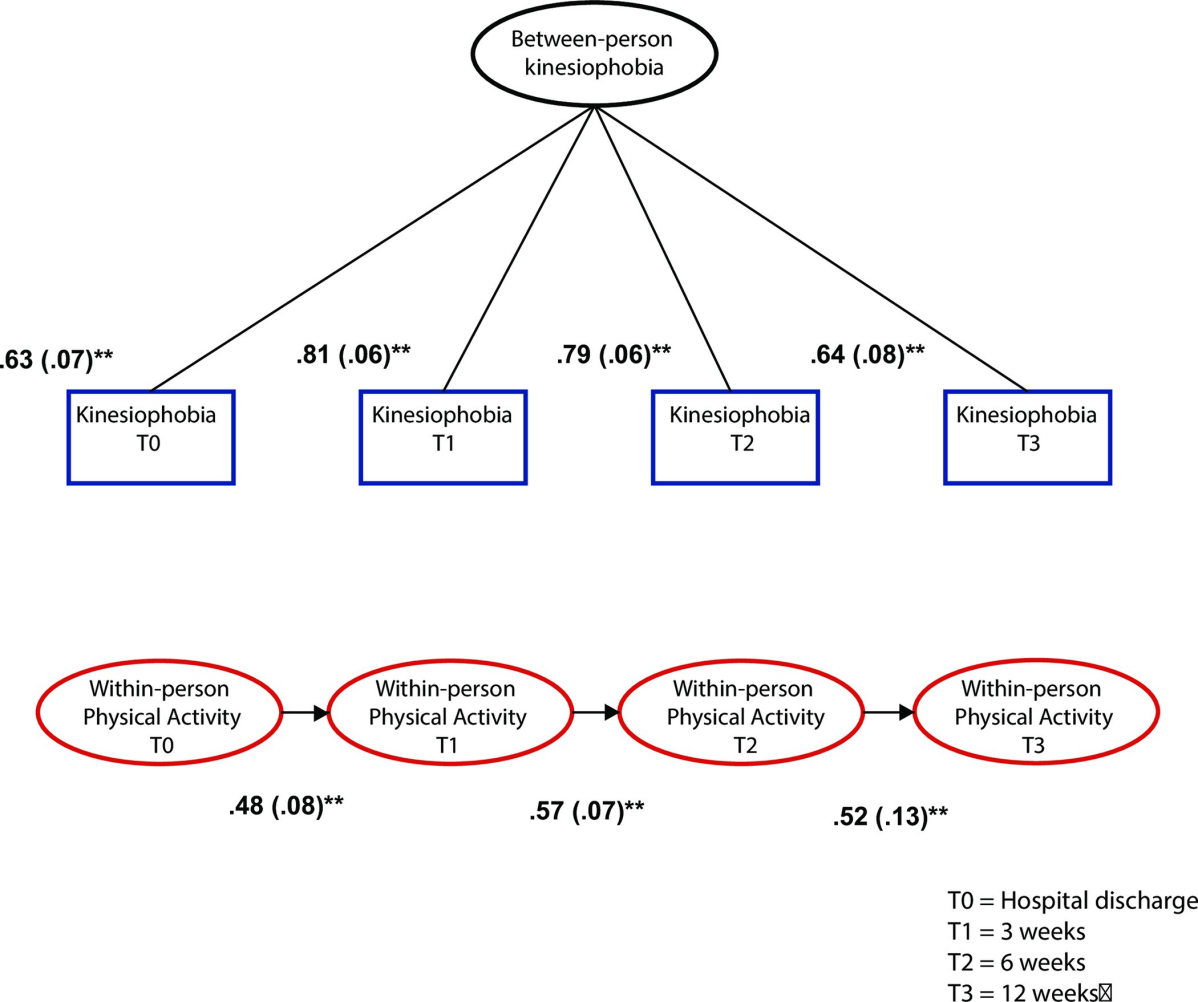
Random intercept model without cross lagged effects.

As described above (see demographics and clinical characteristics) kinesiophobia scores decrease slightly, but significantly. Therefore, after evaluation of the previous model, a new model was proposed by the researchers, where kinesiophobia was modelled as a random intercept (latent variable) and PA as autoregressive variable. This resulted in a model with good model fit (Chi Square: 27.541, P<0.12) **(Table 4)**. This model assumes that kinesiophobia is stable over time and is considered a trait-like construct while PA is considered as a dynamic process with PA levels varying over time. No association was found between objectively measured PA and kinesiophobia. This model is presented in **Fig 5**.

**Table 4:** Random intercept model without cross lagged effects.

	Standardized Beta (95% CI)	S.E.	EST./S.E.
*Random intercept TSK*			
TSK week 1	0.63 (0.49 to 0.78)	0.074	8.511
TSK week 3	0.81 (0.69 to 0.92)	0.059	13.747
TSK week 6	0.79 (0.67 to 0.91)	0.059	13.333
TSK week 12	0.64 (0.49 to 0.78)	0.075	8.539
*Autoregressive effects*			
PA week 3 by PA week 1	0.48 (0.32 to 0.64)	0.082	5.831
PA week 6 by PA week 3	0.57 (0.43 to 0.71)	0.072	7.959
PA week 12 by PA week 6	0.52 (0.26 to 0.77)	0.129	4.003
*Model fit*			
Chi Square = 27.541 P<0.12			
CFI = 0.97			
TLI = 0.96			
DF = 20			

TSK = Tampa Scale for Kinesiophobia

PA = Total physical activity (light + moderate + heavy) (per day/per week)

DF = Degrees of freedom

All effects P < 0.0001

## Discussion

The results of this study show that kinesiophobia and objectively measured PA are not associated with each other in the first 12 weeks after hospital discharge. In addition, we found that kinesiophobia remains stable over time and might be seen as a trait, while light and moderate PA-levels increased over time, especially in the first weeks after hospital discharge.

Concerning the association between kinesiophobia and objectively measured PA, contradictory findings are reported in the literature. A recent study, in patients with low back pain, also shows that kinesiophobia is not associated with objectively measured PA, measured with an accelerometer [13]. In addition, other studies did not find an association between physical capacity measures such as walking endurance and maximal oxygen consumption and kinesiophobia [24–26]. In contrast, Bäck et al., found that patients with high levels of kinesiophobia took fewer steps than those without kinesiophobia 3 to 10 months after cardiac hospitalization [6]. In addition, Bäck et al. reported that cardiac patients with high levels of kinesiophobia avoid high levels of (self-reported) PA [6]. In our study, we found that in the first 12 weeks after hospital discharge, all patients avoid activities with high intensity. An explanation might be that patients are physically active but only avoid certain activities that cause distressing body signals, which are in turn related to kinesiophobia [9]. The results of our study support the absence of an association between kinesiophobia and objectively measured PA in the first 12 weeks after hospital discharge. An explanation for these different findings, might be that all cardiac patients perform less PA in the first weeks after cardiac hospitalization, thereby making discrimination between patients with and without kinesiophobia difficult. Nevertheless, kinesiophobia is consistently associated with disability [13, 26, 27]. In our previously conducted path analysis, we found an association between kinesiophobia, anxiety, educational level and self-efficacy, which are in turn associated with disability [11, 28, 29].

In this study, we used a random intercept cross-lagged panel model to account for between-patient unobserved heterogeneity by decomposing the data into *between-person* and *within-person* variation at various time points. Our final model clearly showed that kinesiophobia is best explained as a stable between-factor and PA-intensity as a dynamic process. This is an important finding and suggests that high levels of kinesiophobia might continue to exist in the months after hospital discharge.

Although the results of our study suggest that kinesiophobia does not impact PA, it is important to target kinesiophobia since patients with kinesiophobia have less self-efficacy, higher levels of anxiety and are less likely to initiate cardiac rehabilitation [9, 10]. Our previous qualitative study, revealed that kinesiophobia is related to negative beliefs about PA (“*By being careful with unnecessary movements I can prevent my heart problems from worsening”* and “*If I tried to be physically active/exercise my heart problem would increase”)*. This study also showed that patients who were exposed to PA, and supported by an informal caregiver during PA in the early phase after hospital discharge, had lower levels of kinesiophobia [9]. Gradual exposure to PA after cardiac hospitalization might improve kinesiophobia. Currently, kinesiophobia, in patients with musculoskeletal complaints, is targeted with exposure in vivo [30–33]. Exposure in vivo, in the form of CR, might help to alleviate fear avoidance beliefs in cardiac patients by gradually exposing patients to PA and thereby altering negative beliefs about PA [33]. Unfortunately, exposure to PA, under the guidance of an experienced physical therapist, is limited to those that are referred to CR. Therefore, adequate referral to CR of those with high levels of kinesiophobia is important.

### Strengths and limitations

This study has several strengths. First, we explored the association between kinesiophobia and PA using a longitudinal design. The longitudinal design allowed us to explore the nature and reciprocity of kinesiophobia and PA after cardiac hospitalization. Second, using the PAM sensor allowed us to measure PA-intensity instead of step-count and thereby giving more detailed insight of PA-patterns in the first 12 weeks after hospital discharge. Third, the RI-CLPM in which we decomposed our data in a *between* and *within* part, enabled us to capture individual fluctuations and account for unobserved heterogeneity.

Some aspects of this study need consideration. First, in this study we investigated the relation between kinesiophobia measured with the TSK-NL Heart. Although the TSK-NL Heart has been found reliable and valid, it might not capture the whole construct of kinesiophobia [34]. The results of our previously conducted qualitative study suggest that kinesiophobia is related to distressing body signals, inconsistent information and passive coping style [9]. Future studies should therefore consider testing the association of PA measures with other fear-related constructs such as: the cardiac anxiety questionnaire (CAQ) [35] or fear avoidance beliefs questionnaire (FABQ) [36]. Second, patients were included in this study at hospital discharge. In the first phase after hospital discharge, patients with no kinesiophobia also might have low PA-levels, which makes discrimination between patients difficult. In addition, the original PA-levels before hospital were unknown. It is therefore difficult to determine if PA-levels were influenced by kinesiophobia. However, this study does show that improvements in PA-levels are not associated with improved levels of kinesiophobia. Future studies should assess PA-levels before hospital admission, changes in PA and kinesiophobia scores over a longer times period. Third, a substantial proportion of patients (38.3%) dropped out of this study which might have led to a selection of patients and might have influenced our findings. It’s unknown of those that dropped out of this study had higher levels of kinesiophobia. Fourth, although we chose to include a heterogenous sample, all patients were discharged after an, acute or elective, interventional procedure. We chose not to include surgical patients since their PA patterns after hospitalization differ from our population. Nevertheless, a variety of TSK-scores and PA-levels were found in our sample and was therefore deemed sufficient to explore the association between kinesiophobia and PA. Future studies should also evaluate the use of graded exposure to improve fear avoidance beliefs in patients with kinesiophobia in studies with a randomized design.

## Conclusion

Kinesiophobia is not associated with objectively measured physical activity in the first 12 weeks after hospital discharge. Levels of kinesiophobia remained stable during this study. Physical activity, on the other hand, is a dynamic process in time. In the first 12 weeks after hospital discharge light and moderate levels of PA fluctuated while high levels of PA were avoided by patients. Future studies should investigate the association between kinesiophobia and PA, using different anxiety measures and over a longer time period.
